# Identification and elucidation of cross talk between SLAM Family Member 7 (SLAMF7) and Toll-like receptor (TLR) pathways in monocytes and macrophages

**DOI:** 10.1038/s41598-023-37040-0

**Published:** 2023-07-07

**Authors:** Uyory Choe, Quynhchi Pham, Young S. Kim, Liangli Yu, Thomas T. Y. Wang

**Affiliations:** 1 Department of Nutrition and Food Science, University of Maryland, College Park, MD 20742 USA; 2 U.S. Department of Agriculture, Agricultural Research Service, Beltsville Human Nutrition Research Center, Diet, Genomics and Immunology Laboratory, Beltsville, MD 20705 USA; 3 Cancer Prevention Science Branch, Division of Cancer Prevention, NCI, Rockville, MD 20850 USA

**Keywords:** Toll-like receptors, Immunology

## Abstract

To further elucidate the expression, regulation and function of Signaling Lymphocytic Activation Molecule Family (SLAMF) protein members in human monocytes and macrophages. Un-differentiated monocytic THP-1 cell (u-THP-1) and differentiated THP-1 macrophage (d-THP-1) were used as culture models in the study. Responses of cells to the differentiation agents phorbol ester (25 ng/ml) and TLR (Toll-like receptor) ligands were assessed. RT-PCR and Western blot analysis were used to determine mRNA and protein level. Pro-inflammatory cytokine mRNA expression levels and phagocytosis were used as functional markers. Data analyzed using t-test, one-way or two-way ANOVA followed by post hoc test. SLAMFs were differentially expressed in THP-1 cells. Differentiation of u-THP-1 to d-THP-1 led to significantly higher SLAMF7 mRNA and protein levels than other SLAMF. In addition, TLR stimuli increased SLAMF7 mRNA expression but not protein expression. Importantly, SLAMF7 agonist antibody and TLR ligands synergistically increased the mRNA expression levels of IL-1β, IL-6 and TNF-α, but had no effect on phagocytosis. SLAMF7 knocked-down in d-THP-1 significantly lowered TLR-induced mRNA expressions of pro-inflammatory markers. SLAM family proteins are differentially regulated by differentiation and TLRs. SLAMF7 enhanced TLR-mediated induction of pro-inflammatory cytokines in monocytes and macrophages but not phagocytosis.

## Introduction

Inflammatory pathways play a vital role in response to many insults such as injury, infection, trauma^1^ and are regulated by immune cells including monocytes and macrophages. During inflammation, monocytes serve as immune effector cells and are equipped with chemokine receptors and adhesion receptors for the role^2^. Monocytes produce pro-inflammatory cytokines such as IL-1β, IL-6, CCL2 and TNF-α, and remove cell debris and pathogens through phagocytosis. Additionally, monocytes are recruited to the site of inflammation in a tissue where they can be differentiated into macrophages^3^. This differentiation process is important since production of pro-inflammatory cytokines such as IL-1β, IL-6, CCL2 and TNF-α by macrophages is critical for an acute inflammatory response^4^. Macrophages also engulf debris, foreign substances, microbes, and cancer cells through phagocytosis. In addition, macrophages play a critical role in innate immunity and help initiate adaptive immunity by recruiting other immune cells such as the lymphocytes^5^.

The SLAM family proteins are a group of type I transmembrane receptors present in immune cells such as monocytes, macrophages, natural killer (NK) cells, CD8+ T lymphocytes, B lymphocytes and mature dendritic cells^6,7^. The SLAMF receptors currently have 9 members with different names in the literature, which include SLAMF1 (SLAM or CD150), SLAMF2 (CD48), SLAMF3 (Ly-9 or CD229), SLAMF4 (2B4 or CD244), SLAMF5 (CD84), SLAMF6 (Ly108 in mice, NTB-A or SF2000 in humans), SLAMF7 (CRACC, CD319 or CS1), SLAMF8 (CD353 or BLAME) and SLAMF9 (SF2001 or CD84H)^8–11^. The SLAMF receptors are important immunomodulatory receptors and have various functions including T cell activation^12–14^, Th2 cytokine production^13–23^, NK- and CD8+ T cells mediated cytotoxicity^24,25^. In general, SLAMF receptors are self-ligand i.e. each SLAM protein recognizes itself to trigger down-stream signaling pathways^26^. This interaction allows different immune cell types to interact with each other through SLAMF receptors^26^. One exception is SLAMF4, which recognizes SLAMF2 and vice versa^26^. The SLAMF receptors are usually composed of two immunoglobulin (Ig) like domains, one variable (V) like domain and one constant 2 (C2) like domain^27^; except SLAMF3 which has four Ig-like domains with two repeated patterns of V-like and C2-like domains^27^. The signal transduction of SLAMF receptors is mediated through signaling lymphocyte activation molecule-associated protein (SAP) adaptors. The SAP adaptors are comprised of SH2 Domain Containing 1A (SAP), SH2 Domain Containing 1B (EAT-2) and EAT-2-related transducer (ERT). SAP adaptors are small proteins that consist of a single Src homology 2 (SH2) domain. Through the SH2 domain, the SAP adaptors interact with immunoreceptor tyrosine-based motifs (ITSMs) in the cytoplasmic domain of SLAMF receptors. Among SAP adaptors, EAT-2 appears to be receptor-specific. For example, in human NK-cells, SLAMF7 recruits exclusively EAT-2 but not SAP^27^. SLAMF receptors can induce signals through interaction with SAP adaptors to various downstream effectors such as nuclear factor of activated T-cells (NFAT), nuclear factor kappa-light-chain-enhancer of activated B cells (NF-κB) and vav guanine nucleotide exchange factor 1 (Vav1) to exert their biological activities^28^. However, some exceptions exist. For example, SLAMF2 is an atypical SLAMF member that has no cytoplasmic region and thus lacks type I transmembrane glycoproteins but has a glycosylphosphatidylinositol membrane anchor^27^. SLAMF8 and SLAMF9 have a short cytoplasmic tail that lacks the ITSM, similar to SLAMF2, and have no known ligands^27^. Although many studies have been reported for SLAMF receptors in T- and NK-cells^9,26,29^, the literature on the function of SLAMF receptors in monocytes and macrophages remain scarce.

Existing literature suggests that the SLAMF proteins and TLRs appear to interact with each other^30,31^. The TLRs are pattern recognition receptors present in immune cells and play an important role as a defense mechanism against bacterial and viral infection^32^. For examples, the gram-negative bacterial cell wall component, LPS, can bind to TLR4. Flagellin, the primary protein component of the flagellar filament in bacteria, can bind to TLR5. Also, bacterial lipoprotein can bind to TLR2/6 and TLR1/2 and synthetic lipopeptides such as Pam2CSK4 and Pam3CSK4 are often used to investigate this pathway^33,34^. When ligands bind to TLRs, signal transduction through transcriptional factors such as NF-κB and AP-1 are activated. Activation of NF-κB and AP-1 induce pro-inflammatory cytokines (TNF-α, IL-1β and IL-6) and chemokines (CCL2 and CXCL8) leading to inflammatory responses which include endothelial cell activation, neutrophil granulocyte activation, fever induction, acute phase protein synthesis, and B lymphocytes proliferation^35^. Farina and others reported that SLAMF1 can be induced by engagement of TLR2, 4 and 5^30^. Sintes et al.^31^ reported that SLAMF5 modulate signaling pathways downstream of TLR4. LPS-stimulated secretion of two pro-inflammatory cytokines (IL-6 and TNF-α) were significantly reduced when RAW-264.7 macrophage cells were transfected with SLAMF5 siRNA^31^. Hence, cross talk between the SLAMF and TLR pathways exist but the precise relationship between specific SLAMF receptors and TLRs remain unclear.

In addition to TLR pathways, the differentiation of monocytes to macrophages may play a critical role in SLAMF protein regulation. Previously, our lab found that SLAMF5 (CD84) mRNA as well as protein levels were up-regulated when phorbol 12-myristate 13-acetate (PMA) was used to induce differentiation of u-THP-1 cells (monocytes) to d-THP-1 cells (macrophages)^36^. Therefore, it is possible that other SLAMFs may also be subjected to similar regulation as SLAMF5, but this conjecture warrants further elucidation.

Recently, SLAMF7 also received much attention due to their high expression in multiple myeloma and therefore could be explored as a potential therapeutic target^37^. This led to the development of Elotuzumab, a novel humanized immunoglobulin G1 (IgG1) monoclonal antibody which targets SLAMF7 on myeloma cancer cell. Elotuzumab has recently been approved for the treatment of relapsed or refractory multiple myeloma in combination with lenalidomide and dexamethasone^38^. The mechanisms appeared to involve activation of NK cell as well as activation of macrophage phagocytosis to eradicate tumor cells^39^. In addition, SLAMF7-mediated macrophage phagocytosis was also reported to be involved in anti-CD47-induced phagocytosis of hematopoietic tumor cells^40^. This effect appeared to be SAP-independent^40^. Finally, SLAMF7 is shown to play a significant role in macrophage-related inflammatory diseases such as rheumatoid arthritis, Crohn’s disease, and COVID-19 infection^41^. However, the role of SLAMF7 and other SLAMs in normal inflammatory processes such as phagocytosis in monocytes and macrophages are less well known and warrant elucidation.

Questions regarding the role of SLAMF proteins and their mechanisms on regulation especially in monocytes and macrophages remain unclear. In this study, we focus on examining the role of SLAMF members in common inflammatory response, such as the interaction with bacterial products to determine 1) if SLAMF proteins are regulated during the differentiation process from monocytes to macrophages, 2) if SLAMF proteins are regulated by the TLRs in monocytes and macrophages, and 3) if SLAMF proteins modulate TLR stimuli-induced production of cytokines and phagocytosis. We focused on SLAMF 1, 3, 4, 5, 6, 7 excluding SLAMF2, SLAMF8 and SLAMF9 due to their atypical composition i.e. lacking ITSM, from other SLAMF members. Our results indicated that SLAMF proteins were differentially regulated by cellular differentiation and TLRs, and SLAMF7 was involved in enhancing TLR-mediated induction of pro-inflammatory cytokines but not phagocytosis in monocytes and macrophages.

## Materials and methods

### Materials and reagents

Flagellin, lipopolysaccharide (LPS) and phorbol 12-myristate 13-acetate (PMA) were purchased from Sigma-Aldrich (St. Louis, MO, USA). Pam2CSK4 was purchased from TOCRIS Bioscience (United Kingdom). Pam3CSK4 was purchased from InvivoGen (San Diego, CA, USA). siRNA for Non-target and for SLAMF7 were purchased from Dharmacon™ (Lafayette, CO, USA). TRIzol reagent was purchased from Invitrogen Life Technologies (Carlsbad, CA, USA). CD14 blocking antibody (MAB3832) was purchased from R&D systems (Minneapolis, MN, USA). All the primers and TaqMan Fast Universal PCR Master Mix were purchased from Applied Biosystems (Carlsbad, CA, USA). AffinityScript Multi-Temperature cDNA Synthesis kit was purchased from Agilent Technologies (Savage, MD, USA). RPMI 1640, FBS, penicillin, and streptomycin were purchased from GIBCO (Grand Island, NY, USA). Radio immunoprecipitation assay (RIPA) buffer, EDTA, protease inhibitor, and phosphatase inhibitors were purchased from Thermo Fisher Scientific (Halethorpe, MD, USA). BCA protein assay kit was purchased from Pierce (Rockford, IL, USA). SLAMF7 primary antibody (Cat#: sc-53576) for Western blots was purchased from Santa Cruz Biotechnology, Inc (Dallas, TX, USA). IRDye®800CW secondary antibody (P/N: 925-32210) was purchased from LI-COR biosciences (Lincoln, NE, USA). Phagocytosis assay kit was purchased from Cell Biolabs, Inc (San Diego, CA, USA). Experiments related to SLAMF7 activation were performed using SLAMF7 Antibody (OTI1F1) (Cat#:NBP2-45868) from Novus Biologicals, LCC (Centennial, CO, USA) and IgG1 control (Cat#:sc-52003) from Santa Cruz Biotechnology, Inc (Dallas, TX. USA).

### Cell culture and differentiation of monocyte to macrophage

Both u-THP-1 and Jurkat (American Type Culture Collection, ATCC, Manassas, VA, USA) were maintained in RPMI 1640 supplemented with 10% FBS and 1% penicillin and streptomycin at 37 °C in a humidified 5% CO_2_ and air environment. For u-THP-1 differentiation (to d-THP-1), u-THP-1 cells (5 × 10^5^ cells/ml) were seeded in 6-well plate or T-175 flask in the presence of 25 ng/ml of phorbol 12-myristate 13-acetate (PMA) for 48 h, covered with aluminum foil. The Jurkat cells (3 × 10^5^ cells/ml) were seeded in 6-well plate.

### Effects of TLRs activation on SLAM family gene and protein expression in u-THP-1 and d-THP-1

For mRNA expression levels, u-THP-1 and d-THP-1 cells were stimulated with four different TLR activators including LPS (10 ng/ml), Flagellin (20 ng/ml), Pam2CSK4 (100 ng/ml), or Pam3CSK4 (25 ng/ml) for 4 h. For western blot protein analysis, u-THP-1 and d-THP-1 cells were stimulated for 24 h as previously described^36^.

### Knock-down of SLAMF7 expression in d-THP-1 using siRNA against SLAMF7

d-THP-1 were seeded at 5 × 10^5^ cells/2 ml/well in a 6-well plate. ON-TARGETplus SMARTpool Human SLAMF7 siRNA or ON-TARGETplus Non-target siRNA at 25 nM concentration and HiPerFect Transfection Reagent (Qiagen, Louisville, KY, USA) were used for SLAMF7 knockdown experiments according to the manufacturer’s protocol. Briefly, the siRNA/HiPerFect complex was made and added dropwise to PMA (25 ng/ml) activated THP-1. The whole cell/complexes solution was incubated for 6 h, then additional fresh cell culture media in the presence of 10% FBS and 1% Pen/Strep was added to each well. Plates were then incubated for 48 h to facilitate transfection followed by with/without LPS incubation for an additional 4 h. After LPS treatment, cells were harvested for RNA isolation. Relative mRNA levels for SLAMF7, IL-1β, IL-6, TNF-α, and CCL2 were determined using RT-PCR as described below.

### Total RNA isolation, cDNA synthesis, and Real-time PCR

Total RNA was isolated by using the TRIzol reagent as previously described^36^. Real-time PCR was used for quantifying changes in relative mRNA levels. 1 µg of total RNA was used for cDNA synthesis using the AffinityScript Multi Temperature cDNA Synthesis kit according to the manufacturer’s protocol. Real-time PCR was performed using the TaqMan Fast Universal PCR Master Mix according to the previously published protocol on ViiA7 real-time PCR system following the manufacturer’s protocol^36^. For the determination of relative mRNA levels, TATA binding protein (Tbp) was used as housekeeping gene for normalization. Relative expression value was generated using Ct method as described by the manufacturer's protocol. The following TaqMan Gene Expression Assays (Thermo Fisher Scientific, Waltham, MA) PCR primer/probe sets were used: SLAMF1(Hs00234149_m1), SLAMF3 (Hs03004331_m1), SLAMF4 (Hs00175568_m1), SLAMF5 (Hs01547121_m1), SLAMF6 (Hs00372941_m1), SLAMF7 (Hs00221793_m1), IL-1b (Hs01555410_m1), IL-6 (Hs00985689_m1), CCL2 (Hs00234140_m1), and TNF-aα (Hs00174128_m1).

### Western blot analysis

Western analysis of protein expression was conducted as described previously^36^. Briefly, after treatments cultured u-THP-1 cells were harvested by centrifugation at 400 RCF for 5 min, cell pellets were washed twice with cold PBS and centrifuge at 400 RCF for 5 min, supernatant removed. Cell pellets were immediately mixed with 100 μL of radio immunoprecipitation assay (RIPA) buffer containing EDTA, protease and phosphatase inhibitors. For d-THP-1 cells, cells were washed twice with cold PBS, harvested by scraping and centrifuged at 400 RCF for 5 min with supernatants removed to obtain final cell pellets. Cell pellets were immediately mixed with 100 μL of radio immunoprecipitation assay (RIPA) buffer containing EDTA, protease and phosphatase inhibitors. Lysates were sonicated three times for 10 s and centrifuged at 400 RCF for 3 min at 4 °C. The protein concentrations in the lysates were determined using a BCA protein assay kit. Routinely, 20 µg of protein per lane was used for electrophoresis separation. The proteins were detected and quantified using the LI-COR Odyssey CLx (LI-COR, Lincoln, NE, USA) Infrared Imager according to manufacturer’s procedure^36^. Full blot digital images of which the cropped images in the figures derived from are in Supplement Figure WB1–6. Two different digital exposures for the images are provided in the supplements. Some membranes were cut as other part of membrane was used for other experiments.

### SLAMF7 activation in u-THP-1 and d-THP-1 using antibody against SLAMF7

u-THP-1 and d-THP-1 (5 × 10^5^ cells/ml) cells were seeded in 6-well plate as previously described^36^ and 10 µg/ml of control IgG_1_ (Cat#:sc-52003) or antibody against SLAMF7 (Cat#:NBP2-45868) was added and incubated for 15 min. After 15 min of incubation, LPS (10 ng/ml), Flagellin (20 ng/ml), Pam2CSK4 (100 ng/ml) or Pam3CSK4 (25 ng/ml) was added and incubated for another 4 h.

### Phagocytosis assay

In 24-well plate, 400 µl of u-THP-1 (5 × 10^5^ cells/ml) cells in the presence of PMA were seeded in each well. Phagocytosis assay kit (Cell Biolabs Inc., San Diego, CA) was used according to the manufacturer’s protocol. Briefly, IgG_1_ (Cat#:sc-52003) or SLAMF7 agonist antibody (10 µg/ml) (Cat#:NBP2-45868) was added to the cell cultures and incubated for 15 min followed by addition of 40 µL of an *E. coli* suspension from the kit. Negative control (without *E. coli*) was also included. Plate was incubated for another 6 h. After incubation, cells in the wells were washed with cold serum-free RPMI medium, followed by addition of a fixation solution and a blocking and permeabilization solution with washes between each step and addition of substrate followed by a stop solution. Absorbance (405 nm) was measured at five different time points (0, 5, 10, 15 and 30 min) using SpectraMax M5 microplate reader (Molecular Devices, San Jose, CA).

### Statistical analysis

GraphPad Prism (Prism 9, GraphPad Software Inc., La Jolla, CA, USA) was used for statistical analysis^42^. Each experiment was performed in triplicate. Data were expressed as mean ± SD. For two groups comparison, t-test was used. For multiple groups either one-way or two-way ANOVA followed by post-hoc test (Tukey test) was used depending on experimental design. In all cases, *p* ≤ 0.05 was considered significant.

## Results

### Expression of SLAMF mRNAs and proteins in the human THP-1 monocytes, THP-1 macrophages and Jurkat cells

**Figure 1:**
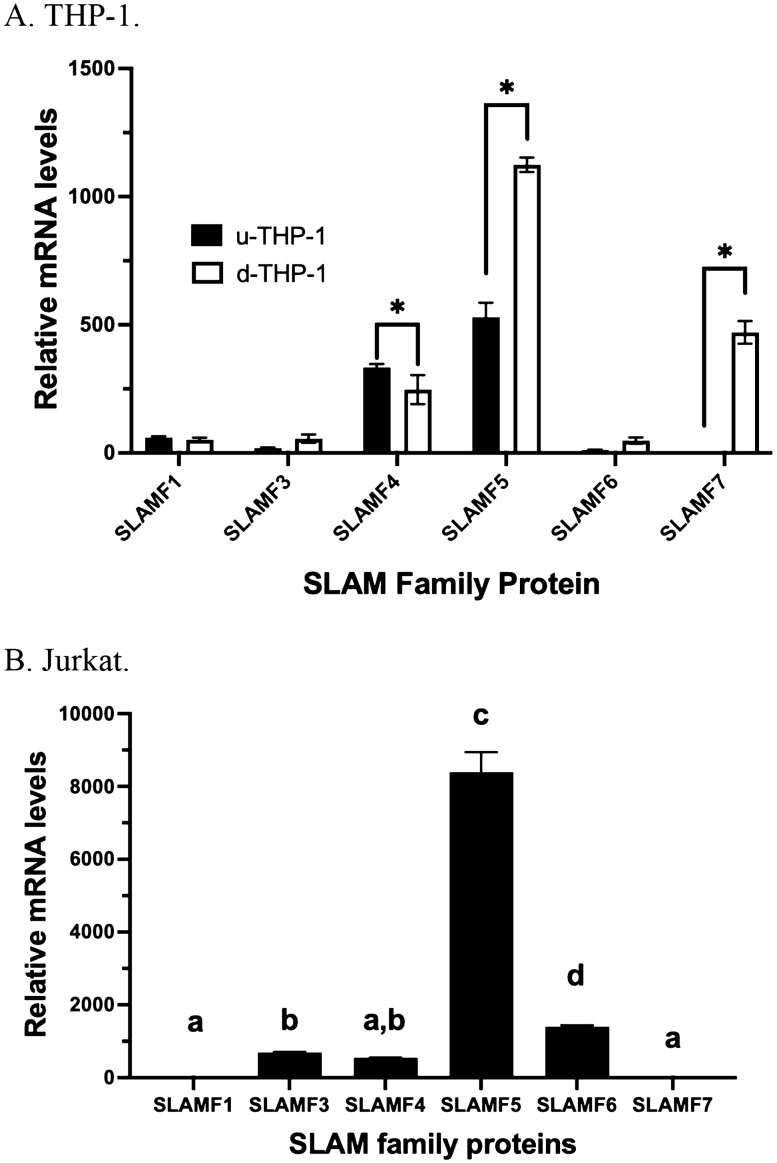
Comparison of SLAMF protein mRNA levels in monocytes (u-THP-1), macrophages (d-THP-1) and Jurkat cells. Cells were cultured and SLAMF protein mRNA levels were determined using real-time PCR. Results are expressed as mean ± SD (n = 3). (**A**) Comparison of SLAMF expression in u-THP-1 and d-THP-1 cells. SLAMF mRNA levels were normalized to SLAMF7 mRNA levels in the u-THP-1 monocytes. Bars with asterisk indicate significant difference to the un-treated control at *p* ≤ 0.05. (**B**) SLAMF protein mRNA levels in Jurkat cells. SLAMF mRNA levels were normalized to SLAMF7. Bars with different letter indicate significant difference at p < 0.05.

**Figure 2:**
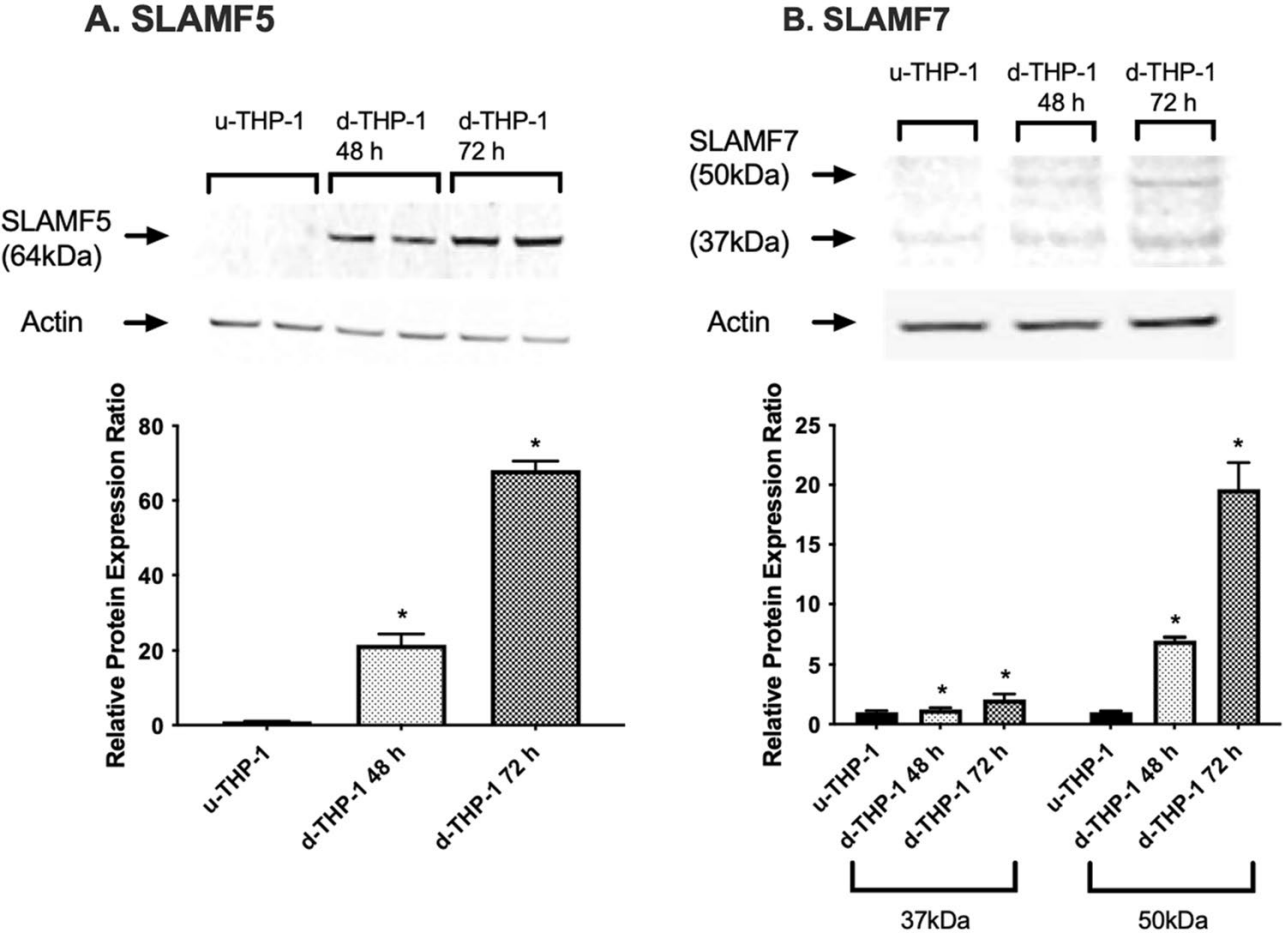
Western blot analysis of SLAMF5 and SLAMF7 protein expression in monocytes (u-THP-1) and macrophages (d-THP-1). Cells were cultured and SLAMF protein levels were determined by Western blot analysis. The proteins were detected and quantified using the LICOR ODYSSEY® CLx (LiCOR, Lincoln, NE, USA) Infrared Imager according to manufacturer’s procedure^37^. Results are expressed as mean ± SD (n = 3). Bars with asterisk indicate significant difference to that of u-THP-1 monocytes (0 time) at *p* ≤ 0.05. (**A**) SLAMF5. Molecular weight of SLAMF5 is 64 kDa. (**B**) SLAMF7. Native molecular weight of SLAMF7 is 37 kDa and glycosylated form is 50 kDa.

We first examined the relative expression of SLAMF mRNAs in human THP-1 cells. As shown in Fig. 1A, SLAMF4 and SLAMF5 are the most highly expressed mRNA in SLAMF proteins in u-THP-1 while other SLAMF members’ expressions were minimal. Interestingly, in d-THP-1, SLAMF5, SLAMF7 were highly expressed, followed by SLAMF4 as the third most highly expressed SLAMF member (Fig. 1A). To further elucidate specificity of SLAMF protein expression in different immune cells, Jurkat cell, a lymphocyte cell model, was also queried for SLAMF as comparison to macrophage/monocyte. In contrast to THP-1 cells, in Jurkat cells (Fig. 1B), SLAMF5 appeared to be the most highly expressed SLAMF protein. In contrast to THP-1 macrophage, SLAMF7 mRNA expression was minimal in Jurkat cells. Also, a significant up-regulation of SLAMF5, SLAMF7 but not SLAMF4 mRNA expression levels were detected during the PMA-induced differentiation of u-THP-1 to d-THP-1. The up-regulated mRNA expression levels of SLAMF5 and 7 were confirmed at the protein level (Fig. 2). In contrast to SLAMF5, two immune-detectable SLAMF7 bands were observed. A 37 kDa and a 50 kDa, representing the original molecular weight of SLAMF7 and its presumable glycosylated form^38^, respectively. PMA induction of THP-1 cells led to time-dependent increase in both immune-detectable SLAMF5 and SLAMF7 proteins (Fig. 2).

### Effect of TLRs activation on SLAMF mRNA levels in monocytes and macrophages

**Figure 3:**
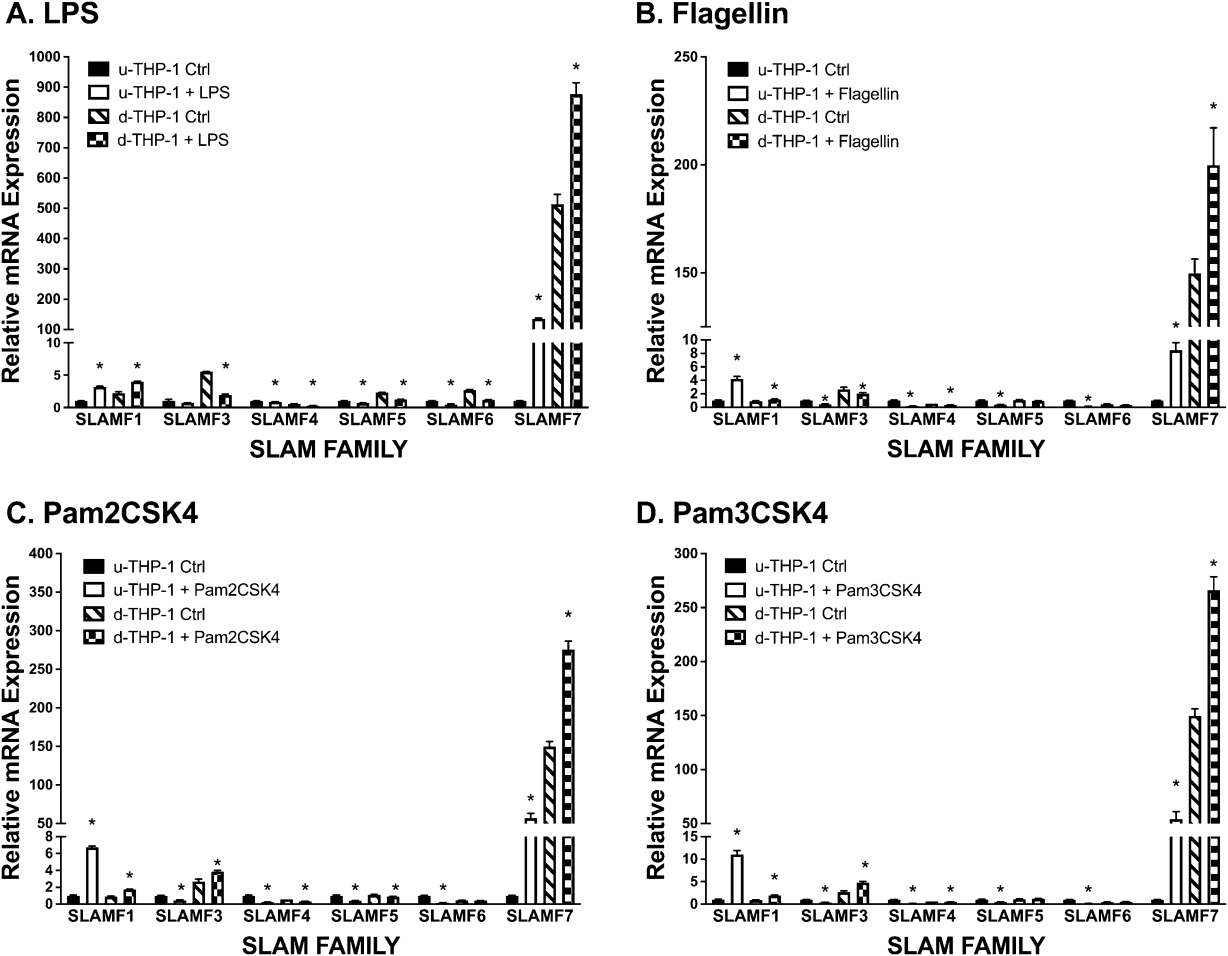
Effects of TLR activation on SLAMF mRNA levels in monocytes (u-THP-1) and macrophages (d-THP-1). Cells were cultured, treated with TLR ligands and SLAMF protein mRNA levels were determined using real-time PCR. Results normalized to respective gene’s u-THP-1 control and are expressed as mean ± SD (n = 3). Bars with asterisk indicate significant difference to the un-treated control at *p* ≤ 0.05. (**A**) LPS. (**B**) Flagellin. (**C**) Pam2CSK4. (**D**) Pam3CSK4.

We next determined the effects of TLR activation on SLAMFs given the role of TLR in host’s immune responses. SLAMF protein mRNA expression levels were evaluated when u-THP-1 and d-THP-1 cells were activated with specific TLR ligands. As shown in Fig. 3, only SLAMF7 was significantly induced by all four TLR stimuli including LPS, Flagellin, Pam2CSK4 and Pam3CSK4. Compared to SLAMF7 mRNA expression levels, other SLAMF protein expression levels were minimal.

Since we previously published work on expression, regulation and immune-related roles of SLAMF5^36^ and given the uniqueness of SLAMF7 expression, regulation patterns, we focused on SLAMF7 for follow-up analysis.

### Characterization of LPS responses of SLAMF7 in monocyte (u-THP-1) and macrophage (d-THP-1)

#### CD14 dependency of LPS-induced increase in SLAMF7 mRNA levels

**Figure 4:**
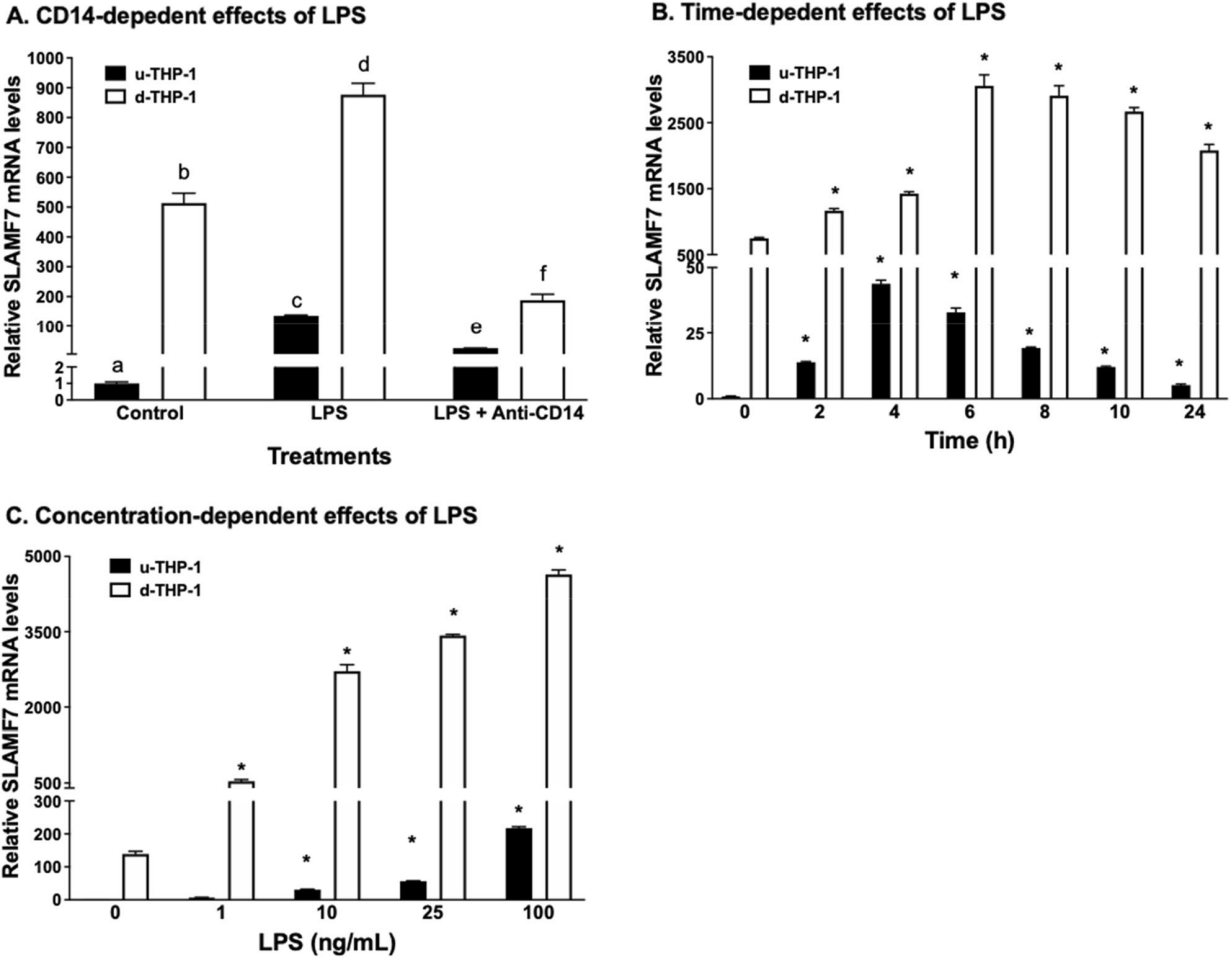
Characterization of LPS induction of SLAMF7 mRNA in monocytes (u-THP-1) and macrophages (d-THP-1). Cells were cultured, treated with LPS and SLAMF protein mRNA levels were determined using real-time PCR. Testing of CD14 dependency was conducted in the presence or absence of anti-CD14 antibody. Results are expressed as mean ± SD (n = 3). Bars with different letters indicate significant difference at *p* ≤ 0.05. Bars with asterisk indicate significant difference to the un-treated control at *p* ≤ 0.05. (**A**) CD14-dependent signaling. (**B**) Time-dependent effects of LPS. (**C**) Concentration-dependent effects of LPS.

Because TLR4 can be activated through CD14-dependent or independent pathway, we further characterized the CD14 dependency of the TLR4 ligand LPS induction of SLAMF7 mRNA levels. The pathway of LPS-induced SLAMF7 mRNA expressions in u-THP-1 and d-THP-1 was evaluated using CD14 blocking antibody. Blocking CD14 in both u-THP-1 and d-THP-1 significantly reduced LPS-induced SLAMF7 mRNA expression levels (Fig. 4A).

#### Time-dependent effects of LPS on SLAMF7 mRNA levels

The mRNA expression levels of SLAMF7 in u-THP-1 cells stimulated with LPS peaked at 4 h and then decreased. In d-THP-1 cells, mRNA expression levels of SLAMF7 stimulated with LPS peaked at 6 h and then decreased (Fig. 4B).

#### Concentration dependent effect of LPS on SLAMF7 mRNA levels

LPS induced a concentration dependent increase in SLAMF7 mRNA expression levels in both u-THP-1 and d-THP-1 cells (Fig. 4C). Induction of SLAMF7 mRNA levels in both u-THP-1 and d-THP1 cells was significantly induced by LPS at 1 ng/ml.

#### LPS stimulation affect SLAMF7 at protein level

**Figure 5:**
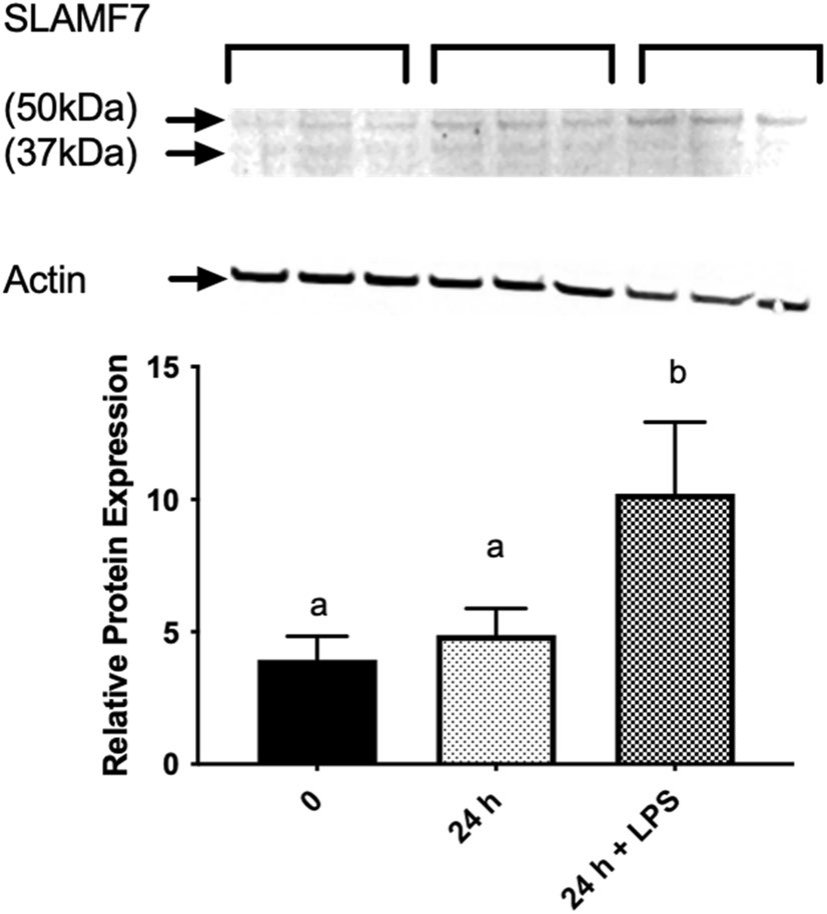
Western blot analysis of SLAMF7 protein expression in macrophages (d-THP-1) in the presence of LPS. Cells were cultured and SLAMF7 protein levels were determined by Western blot analysis. The proteins were detected and quantified using the LICOR ODYSSEY® CLx (LiCOR, Lincoln, NE, USA) Infrared Imager according to manufacturer’s procedure^37^. Results are expressed as mean ± SD (n = 3). Different letters indicate significant difference to that of d-THP-1 (0 time) at *p* ≤ 0.05.

We also seek to confirm LPS stimulation of SLAMF7 at the protein level. TLRs activation significantly induced SLAMF7 protein expression (Fig. 5).

### Effects of SLAMF7 siRNA knocked-down in d-THP-1 on LPS-induced cytokines

**Figure 6:**
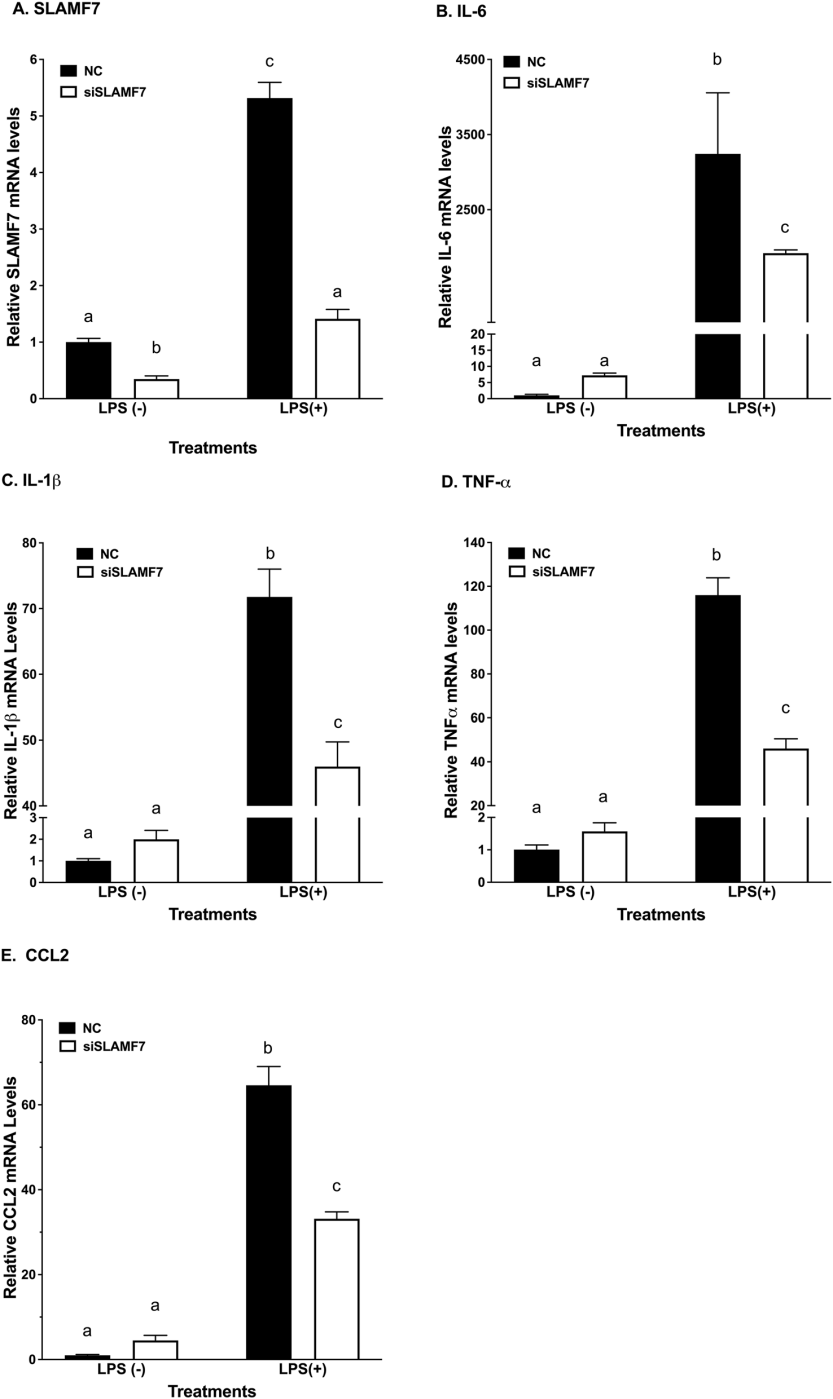
Effects of SLAMF7 siRNA on LPS-induced cytokines mRNA levels. Cells were treated with negative control (NC) or SLAMF7 siRNA (siSLAMF7). Transfected cells were then treated with or without LPS. Total RNA isolated after treatments and marker cytokine mRNA levels were determined using real-time PCR. Results are expressed as mean ± SD (n = 3). Bars with different letters indicate significant difference at *p* ≤ 0.05. (**A**) SLAMF7 (**B**) IL-6 (**C**) IL-1β (**D**) TNF-α (**E**) CCL2.

The unique expression and regulation of SLAMF7 in macrophage led us to evaluate the functional roles of SLAMF7 in monocyte/macrophage. Using siRNA knockdown experiment, SLAMF7 knock-down in d-THP-d significantly reduced SLAMF7 mRNA expression levels with and without LPS stimulation (Fig. 6A). In the presence of LPS, SLAMF7 knock-down in d-THP-1 significantly attenuated LPS-induced mRNA expression levels of pro-inflammatory cytokines including IL-1β, IL-6, CCL2 and TNF-α compared to the non-target control (NC) (Fig. 6B–E).

### Identifying SLAMF7 activating antibody and its effect on TLR stimuli-induction of cytokines

**Figure 7:**
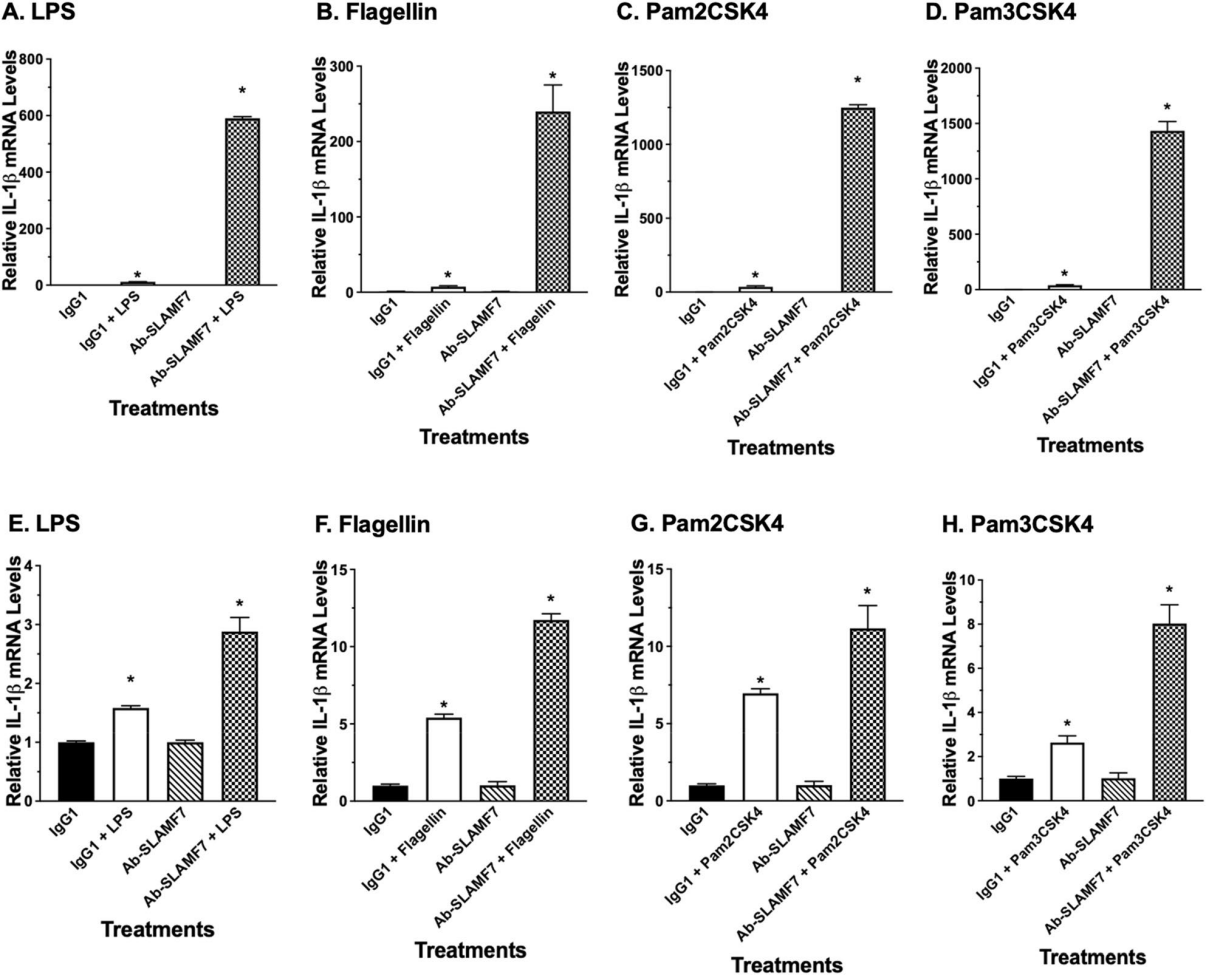
Effects of SLAMF7 antibody on TLR-induced IL-1β mRNA levels. Cells were treated with or without TLR ligands in the presence or absence of SLAMF7 antibody. Total RNA isolated after treatments and IL-1β mRNA levels were determined using real-time PCR. Results are expressed as mean ± SD (n = 3). Bars with asterisk indicate significant difference to the un-treated control at *p* ≤ 0.05. Panel (**A**) LPS (**B**) Flagellin (**C**) Pam2CSK4 (**D**) Pam3CSK4 illustrate effects in u-THP-1. Panel (**E**) LPS (**F**) Flagellin (**G**) Pam2CSK4 (**H**) Pam3CSK4 illustrate effects in d-THP-1.

Based on reported Elotuzumab activating property^38,39^, we seek to identify commercially available antibody that can activate SLAMF7. We found that addition of the SLAMF7 antibody (Cat#:NBP2-45868), but not IgG_1_ control antibody (Cat#:sc-52003), enhanced up-regulation of pro-inflammatory cytokines including IL-1β, IL-6 and TNF-α in u-THP-1 cells by TLR ligands LPS, Flagellin, Pam2CSK4 or Pam3CSK4, (Fig. 7, S1 and S3). Similarly, when d-THP-1 was activated with LPS, Flagellin or Pam3CSK4 in presence of SLAMF7 agonist antibody, enhanced up-regulation of pro-inflammatory cytokines including IL-1β, IL-6 and TNF-α was also observed. In contrast, activation with Pam2CSK4 in the presence of SLAMF7 agonist antibody slightly lowered IL-6 and TNF-α mRNA expression levels compared to its IgG_1_ control (Fig. S1–S3).

### SLAMF7’s effect on macrophages (d-THP-1) phagocytosis activity

**Figure 8:**
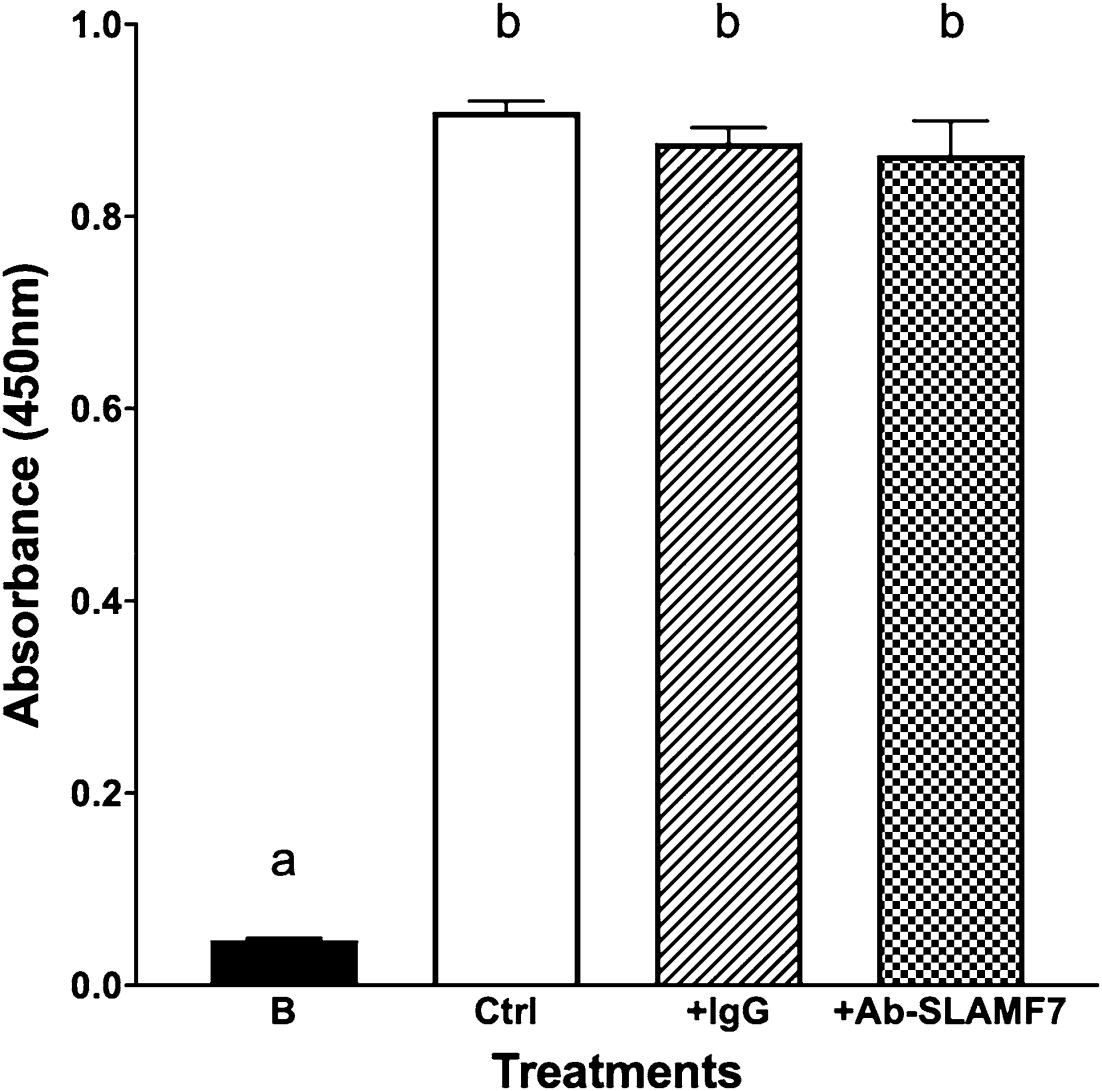
Effects of SLAMF7 antibody on phagocytosis activity of macrophage (d-THP-1). U-THP-1 cells were differentiated to d-THP-1 cells and phagocytosis activity was determined in the presence or absence of control antibody or SLAMF7 antibody. B: Blank (without *E. coli* particle) or negative control, and Ctrl: *E. coli* particles were used as a phagocytosis pathogen or positive control. +IgG: with isotype IgG control added, +Ab-SLAMF7: with antibody against SLAMF7 added. Results are expressed as mean ± SD (n = 3). Bars with different letters indicate significant difference at *p* ≤ 0.05.

In addition to cytokine production, we also asked if SLAMF7 played a role in phagocytotic activity of macrophage. Using the SLAMF7 agonist antibody described above, that enhanced cytokine production, we did not observe an effect of the antibody on d-THP-1 phagocytosis activity as compared to IgG_1_ or a positive control (Fig. 8).

## Discussion

In the present study, several important pieces of information related to SLAMF proteins were identified which included: 1) Identification of crosstalk between SLAMF7 and several selected TLRs in the monocyte/macrophage. 2) SLAMF7 to be the major SLAMF protein regulated in monocyte/macrophage in term of fold changes. 2) Different types of immune cells express different sets of SLAMF protein may indicate functional selectivity. 3) Differentiation and TLR stimuli regulated specific SLAMF members in monocyte/macrophage. 4) Functionally, SLAMF7 activation enhanced selected TLR-induced cytokine responses but not phagocytosis.

The SLAMF receptors are important immunomodulatory receptors in the immune cells. These receptors are known to have a wide spectrum of roles including regulation of cytotoxicity, humoral immunity, autoimmunity, cell survival, lymphocyte development, cell adhesion, invasiveness, and production of cytokines^6–25,43,44^. Moreover, due to having multiple members in the SLAMF receptors, their expression patterns in immune cells such as monocytes and macrophages remained unclear but may be important contributors to their function. Our results indicated that monocyte SLAMF4 and 5 appeared to be the dominant SLAMF in monocytes; which was consistent with previously published reports^24,25^. These results suggested that SLAMF4 and 5 may play a relatively more central role than other SLAMF in mediating monocyte’s function.

Differentiation of monocyte to macrophage significantly altered the expression profile of SLAMF protein; most notably, an induction of SLAMF7 expression. Comparing human T lymphocyte Jurkat cells to THP-1 monocyte/macrophage (Fig. 1B), SLAMF7 showed unique expression in the macrophage, suggesting a specific functional role for this protein in immune responses of macrophages. The biological efficacies specific to SLAMF7 were confirmed from experiments using siRNA against SLAMF7. Transient transfection with SLAMF7 siRNA to knock-down SLAMF7 expression in d-THP-1 significantly reduced LPS activated mRNA expression levels of pro-inflammatory cytokines including IL-1β, IL-6, CCL2 and TNF-α (Fig. 6A–E). These data support SLAMF7 play an enhancing role in mediating the effects of inflammatory stimuli in macrophage. Unfortunately, the efficiency of siRNA for u-THP-1 was too low (data not shown), therefore, experiments in u-THP-1 were not able to be conducted. This data is consistent with reported literature that u-THP-1 cells are notoriously difficult to transfect^45^. Importantly, we identified a SLAMF7 antibody that function as an agonist antibody.

The SLAMF7 antibody (Cat#NBP2-45868), enhanced up-regulation of pro-inflammatory cytokines including IL-1β, IL-6 and TNF-α mRNA by TLR ligands LPS (TLR4), Flagellin (TLR5) and Pam3CSK4 (TLR1/2) (Fig. 7, S1–S3) in both d- and u-THP-1 cells. These data are consistent with a recent report by Simmons et al.^41^ and provide independent verification of an enhanced immune response by SLAMF7 engagement. One exception from our study was observed in Pam2CSK4 (TLR2/6 ligand)-activated d-THP-1 (Fig. S1–S3). In this case, the SLAMF7 agonist antibody and Pam2CSK4 did not synergistically induce IL-6, CCL2 and TNF-α. These results suggested that SLAMF7 may only interact with selective TLR to enhance the immune responses elicit by those TLRs, but more studies are needed to further elucidate the specific differences. In addition, the chemokine CCL2 showed a slightly different mRNA expression levels compared to the other cytokines (Fig. S2) suggesting that selective modulation of specific cytokines/chemokines pathways by SLAMF7 may also exist. Overall, our results from siRNA and agonist antibody supported involvement of SLAMF7 in the regulation of selected TLR-induced pro-inflammatory cytokines/chemokines in u-THP-1 and d-THP-1. This cross talk between SLAMF7 and selected TLR allow for the monocyte/macrophage to mount a concerted robust immune response during infection. Of note, the agonist antibody enhanced the selected TLR-induction of cytokines (Fig. 7) only in the presence of TLR ligands. Hence, SLAMF7 engagement itself is not capable of directly induce cytokines but rather SLAMF7 appeared to play a supporting role in the process. The interaction between TLR and SLAMF7 appeared to be through CD14-related pathway as knock-down of CD14 inhibited the induction by LPS (Fig. 4A).

We also evaluated SLAMF7 biological efficacy in phagocytosis, as phagocytosis is a major mechanism by which monocyte/macrophage remove pathogens and cell debris^46^. However, we did not observed effects of agonist antibody on phagocytosis. These results and the cytokine analysis supported the cross talk between SLAMF7 and TLR pathways that regulates cytokine production but not phagocytosis.

Several other SLAMF were also upregulated during differentiation (Fig. 1A). Therefore, differentiation is apparently the main driver for regulating the SLAMF pathway. Although we have not tested all up-regulated SLAMF pathways, we suspect the SLAMF pathway provide overall promotional effects on host’s immune response through interaction with TLR-associated pathways. SLAMF1, 4 appeared not to be regulated by differentiation or TLR stimuli, therefore may not contribute to enhanced immune responses, such as cytokine production, in the macrophage. However, additional studies are needed to further elucidate the specific role for other SLAMF not examined in this study in monocyte/macrophage.

## Conclusion

Our study indicated that SLAMF receptors are differentially expressed in immune cells. We also provided data to support differentiation from monocytes to macrophages lead to up-regulation of SLAMF7 that promote induction of cytokines by selected TLR ligands in monocytes and macrophages. Overall, our results indicate crosstalk and a coordinated response of selected TLR and SLAMF7-mediated pathways against immune stimuli in monocyte/macrophage exist.

## Supplementary Information

Supplementary Figures.

Supplementary Information 1.

## Data Availability

The datasets used and/or analyzed in the current study are available from the corresponding author on reasonable request.

## Abbreviations

d-THP-1: Differentiated THP-1 macrophage
u-THP-1: Undifferentiated THP-1 monocyte
LPS: Lipopolysaccharide
IL-1β: Interleukin 1 beta
IL-6: Interleukin 6
CCL2: Chemokine ligand 2
TNF-α: Tumor necrosis factor alpha
COX-2: Cyclooxygenase 2
PGE_2_: Prostaglandin E2
IFN-γ: Interferon gamma

## References

[1] Ward PA (1999). The acute inflammatory response and its regulation. Arch. Surg..

[2] Lauvau G, Chorro L, Spaulding E, Soudja SMH (2014). Inflammatory monocyte effector mechanisms. Cell. Immunol..

[3] Kurihara T, Warr G, Loy J, Bravo R (1997). Defects in macrophage recruitment and host defense in mice lacking the CCR2 chemokine receptor. J. Exp. Med..

[4] Yang J, Zhang L, Yu C, Yang X-F, Wang H (2014). Monocyte and macrophage differentiation: Circulation inflammatory monocyte as biomarker for inflammatory diseases. Biomark. Res..

[5] Iwasaki A, Medzhitov R (2010). Regulation of adaptive immunity by the innate immune system. Science.

[6] Boles KS, Mathew PA (2001). Molecular cloning of CS1, a novel human natural killer cell receptor belonging to the CD2 subset of the immunoglobulin superfamily. Immunogenetics.

[7] Bouchon A, Cella M, Grierson HL, Cohen JI, Colonna M (2001). Cutting edge: Activation of NK cell-mediated cytotoxicity by a SAP-independent receptor of the CD2 family. J. Immunol..

[8] Ma CS, Nichols KE, Tangye SG (2007). Regulation of cellular and humoral immune responses by the SLAM and SAP families of molecules. Annu. Rev. Immunol..

[9] Veillette A (2006). Immune regulation by SLAM family receptors and SAP-related adaptors. Nat. Rev. Immunol..

[10] Veillette A, Dong Z, Latour S (2007). Consequence of the SLAM-SAP signaling pathway in innate-like and conventional lymphocytes. Immunity.

[11] Engel P, Eck MJ, Terhorst C (2003). The SAP and SLAM families in immune responses and X-linked lymphoproliferative disease. Nat. Rev. Immunol..

[12] Czar MJ, Kersh EN, Mijares LA, Lanier G, Lewis J, Yap G (2001). Altered lymphocyte responses and cytokine production in mice deficient in the X-linked lymphoproliferative disease gene SH2D1A/DSHP/SAP. Proc. Natl. Acad. Sci. USA.

[13] Cocks BG, Chang C-CJ, Carballido JM, Yssel H, Vries JED, Aversa G (1995). A novel receptor involved in T-cell activation. Nature.

[14] Howie D (2002). The role of SAP in murine CD150 (SLAM)-mediated T-cell proliferation and interferon gamma production. Blood.

[15] Aversa G, Carballido J, Punnonen J, Chang C-CJ, Hauser T, Cocks BG (1997). SLAM and its role in T cell activation and Th cell responses. Immunol. Cell Biol..

[16] Cannons JL, Yu LJ, Hill B, Mijares LA, Dombroski D, Nichols KE (2004). SAP regulates TH2 differentiation and PKC-θ-mediated activation of NF-κB1. Immunity.

[17] Dupre L (2005). SAP controls the cytolytic activity of CD8 T cells against EBV-infected cells. Blood.

[18] Graham DB, Bell MP, Mccausland MM, Huntoon CJ, Deursen JV, Faubion WA (2006). Ly9 (CD229)-deficient mice exhibit T cell defects yet do not share several phenotypic characteristics associated with SLAM- and SAP-deficient mice. J. Immunol..

[19] Howie D, Laroux FS, Morra M, Satoskar AR, Rosas LE, Faubion WA (2005). Cutting edge: The SLAM family receptor Ly108 controls T cell and neutrophil functions. J. Immunol..

[20] Martin M, Romero X, Fuente MADL, Tovar V, Zapater N, Esplugues E (2001). CD84 functions as a homophilic adhesion molecule and enhances IFN-secretion: Adhesion is mediated by Ig-like domain 1. J. Immunol..

[21] Martin M, Valle JMD, Saborit I, Engel P (2005). Identification of Grb2 As a novel binding partner of the signaling lymphocytic activation molecule-associated protein binding receptor CD229. J. Immunol..

[22] Wang N, Satoskar A, Faubion W, Howie D, Okamoto S, Feske S (2004). The cell surface receptor SLAM controls T cell and macrophage functions. J. Exp. Med..

[23] Yusuf I, Kageyama R, Monticelli L, Johnston RJ, Ditoro D, Hansen K (2010). Germinal Center T follicular helper cell IL-4 production is dependent on signaling lymphocytic activation molecule receptor (CD150). J. Immunol..

[24] Nakajima H, Cella M, Langen H, Friedlein A, Colonna M (1999). Activating interactions in human NK cell recognition: The role of 2B4-CD48. Eur. J. Immunol..

[25] Bottino C, Falco M, Parolini S, Marcenaro E, Augugliaro R, Sivori S (2001). Gntb-A, a novel Sh2d1a-associated surface molecule contributing to the inability of natural killer cells to kill Epstein-Barr virus-infected B cells in X-linked lymphoproliferative disease. J. Exp. Med..

[26] Detre C, Keszei M, Romero X, Tsokos GC, Terhorst C (2010). SLAM family receptors and the SLAM-associated protein (SAP) modulate T cell functions. Semin. Immunopathol..

[27] Veillette A (2010). SLAM-family receptors: immune regulators with or without SAP-family adaptors. Cold Spring Harbor Perspect. Biol..

[28] Schwartzberg PL, Mueller KL, Qi H, Cannons JL (2009). SLAM receptors and SAP influence lymphocyte interactions, development and function. Nat. Rev. Immunol..

[29] Cannons JL, Qi H, Lu KT, Dutta M, Gomez-Rodriguez J, Cheng J (2010). Optimal germinal center responses require a multistage T cell: B cell adhesion process involving integrins, SLAM-associated protein, and CD84. Immunity.

[30] Weber MS, Starck M, Wagenpfeil S, Meinl E, Hohlfeld R, Farina C (2004). Multiple sclerosis: Glatiramer acetate inhibits monocyte reactivity in vitro and in vivo. Brain.

[31] Sintes J, Romero X, Salort JD, Terhorst C, Engel P (2010). Mouse CD84 is a pan-leukocyte cell-surface molecule that modulates LPS-induced cytokine secretion by macrophages. J. Leukoc. Biol..

[32] Kawasaki T, Kawai T (2014). Toll-like receptor signaling pathways. Front. Immunol..

[33] Lu Y-C, Yeh W-C, Ohashi PS (2008). LPS/TLR4 signal transduction pathway. Cytokine.

[34] Moresco EMY, Lavine D, Beutler B (2011). Toll-like receptors. Curr. Biol..

[35] Duque GA, Descoteaux A (2014). Macrophage cytokines: Involvement in immunity and infectious diseases. Front. Immunol..

[36] Wang T, Pham Q, Kim Y (2018). Elucidating the role of CD84 and AHR in modulation of LPS-induced cytokines production by cruciferous vegetable-derived compounds indole-3-carbinol and 3,3′-diindolylmethane. Int. J. Mol. Sci..

[37] Guo H, Cruz-Munoz M-E, Wu N, Robbins M, Veillette A (2014). Immune cell inhibition by SLAMF7 is mediated by a mechanism requiring Src kinases, CD45, and SHIP-1 that is defective in multiple myeloma cells. Mol. Cell Biol..

[38] Lonial, S., Dimopoulos, M., Palumbo, A., White, D., Grosicki, S., Spicka, I., Walter-Croneck, A., Moreau, P., Mateos, M. V., Magen, H., Belch, A., Reece, D., Beksac, M., Spencer, A., Oakervee, H., Orlowski, R.Z., Taniwaki, M., Röllig, C., Einsele, H., Wu, K. L., Singhal, A., San-Miguel, J., Matsumoto, M., Katz, J., Bleickardt, E., Poulart, V., Anderson, K. C., Richardson, P., ELOQUENT-2 Investigators. Elotuzumab therapy for relapsed or refractory multiple myeloma. N. Engl. J. Med. 2015;373:621–631@@@. 10.1056/NEJMoa1505654 26035255

[39] Raje N, Longo DL (2015). Monoclonal antibodies in multiple myeloma come of age. N. Engl. J. Med..

[40] Chen J, Zhong M-C, Guo H, Davidson D, Mishel S, Lu Y (2017). SLAMF7 is critical for phagocytosis of haematopoietic tumour cells via Mac-1 integrin. Nature.

[41] Simmons DP, Nguyen HN, Gomez-Rivas E, Jeong Y, Jonsson AH, Chen AF, Lange JK, Dyer GS, Blazar P, Earp BE, Coblyn JS (2022). SLAMF7 engagement superactivates macrophages in acute and chronic inflammation. Sci. Immunol..

[42] Statistical analyses were performed using GraphPad Prism version 9.4.1 for Windows, GraphPad Software. www.graphpad.com

[43] Chuang SS, Kim MH, Johnson LA, Albertsson P, Kitson RP, Nannmark U (2000). 2B4 stimulation of YT cells induces natural killer cell cytolytic function and invasiveness. Immunology.

[44] Kim JR, Horton NC, Mathew SO, Mathew PA (2013). CS1 (SLAMF7) inhibits production of proinflammatory cytokines by activated monocytes. Inflamm. Res..

[45] Schnoor M, Buers I, Sietmann A, Brodde MF, Hofnagel O, Robenek H (2009). Efficient non-viral transfection of THP-1 cells. J. Immunol. Methods.

[46] Cockram TOJ, Dundee JM, Popescu AS, Brown GC (2021). The phagocytic code regulating phagocytosis of mammalian cells. Front. Immunol..

