# Supplementary material for: Identification and elucidation of cross talk between SLAM Family Member 7 (SLAMF7) and Toll-like receptor (TLR) pathways in monocytes and macrophages
Source: Sci Rep. 2023 Jul 7;13:11007. doi: 10.1038/s41598-023-37040-0 (PMC10329007; doi:10.1038/s41598-023-37040-0)
Supplement: Supplementary file 1 — Supplementary Figures. [file 41598_2023_37040_MOESM1_ESM.docx]

Figure S1.


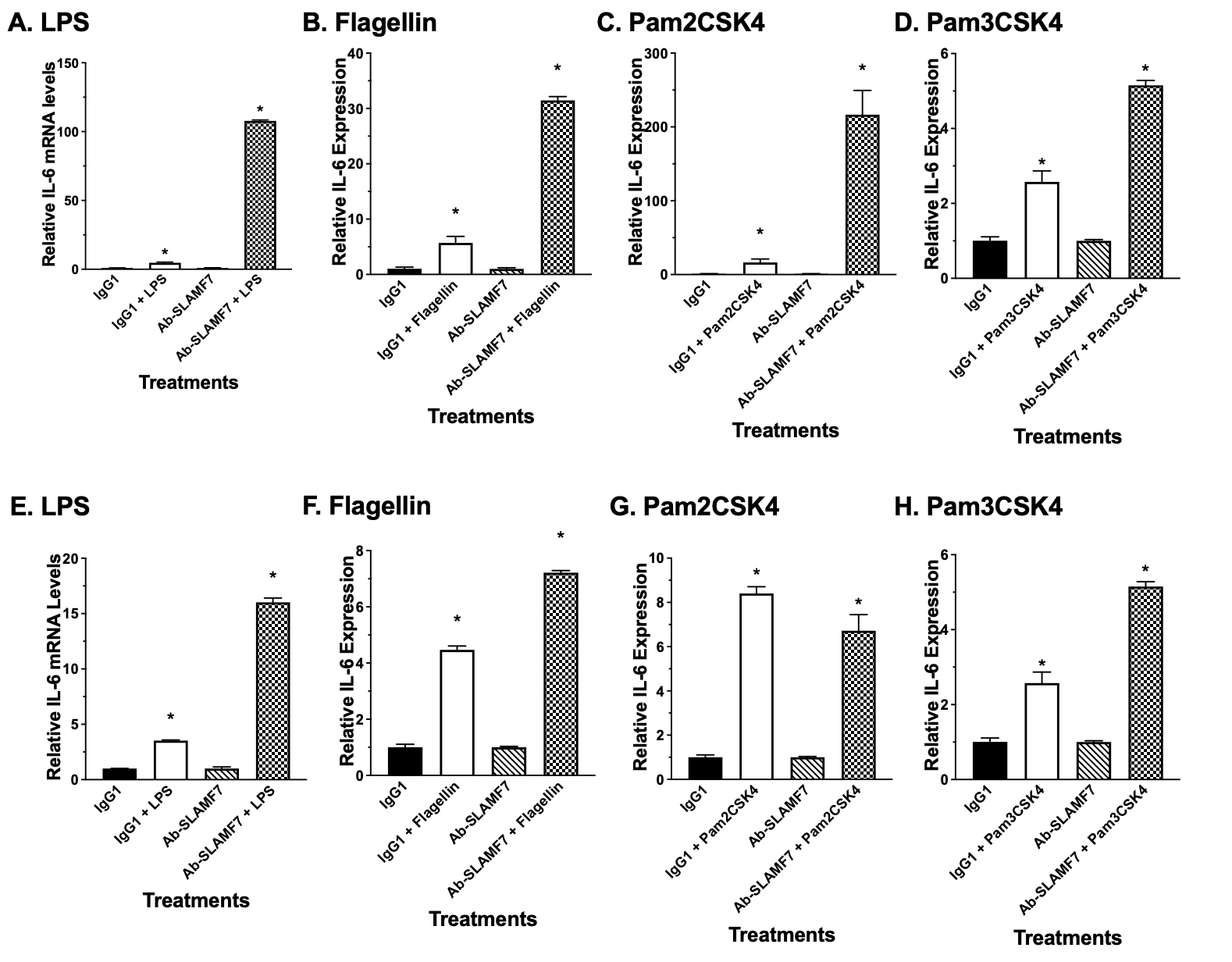


**Fig. S1** Effects of SLAMF7 antibody on TLR-induced IL-6 mRNA levels. Cells were treated with or without TLR ligands in the presence or absence of SLAMF7 antibody. Total RNA isolated after treatments and IL-6 mRNA levels were determined using real-time PCR. Results are expressed as mean ± SD (n = 3). Bars with asterisk indicate significant difference to the un-treated control at *p* ≤ 0.05.  **Panel (A)** LPS **(B)** Flagellin **(C)** Pam2CSK4 **(D)** Pam3CSK4 illustrate effects in u-THP-1. Panel **(E)** LPS **(F)** Flagellin **(G)** Pam2CSK4 **(H)** Pam3CSK4 illustrate effects in d-THP-1.

Figure S2


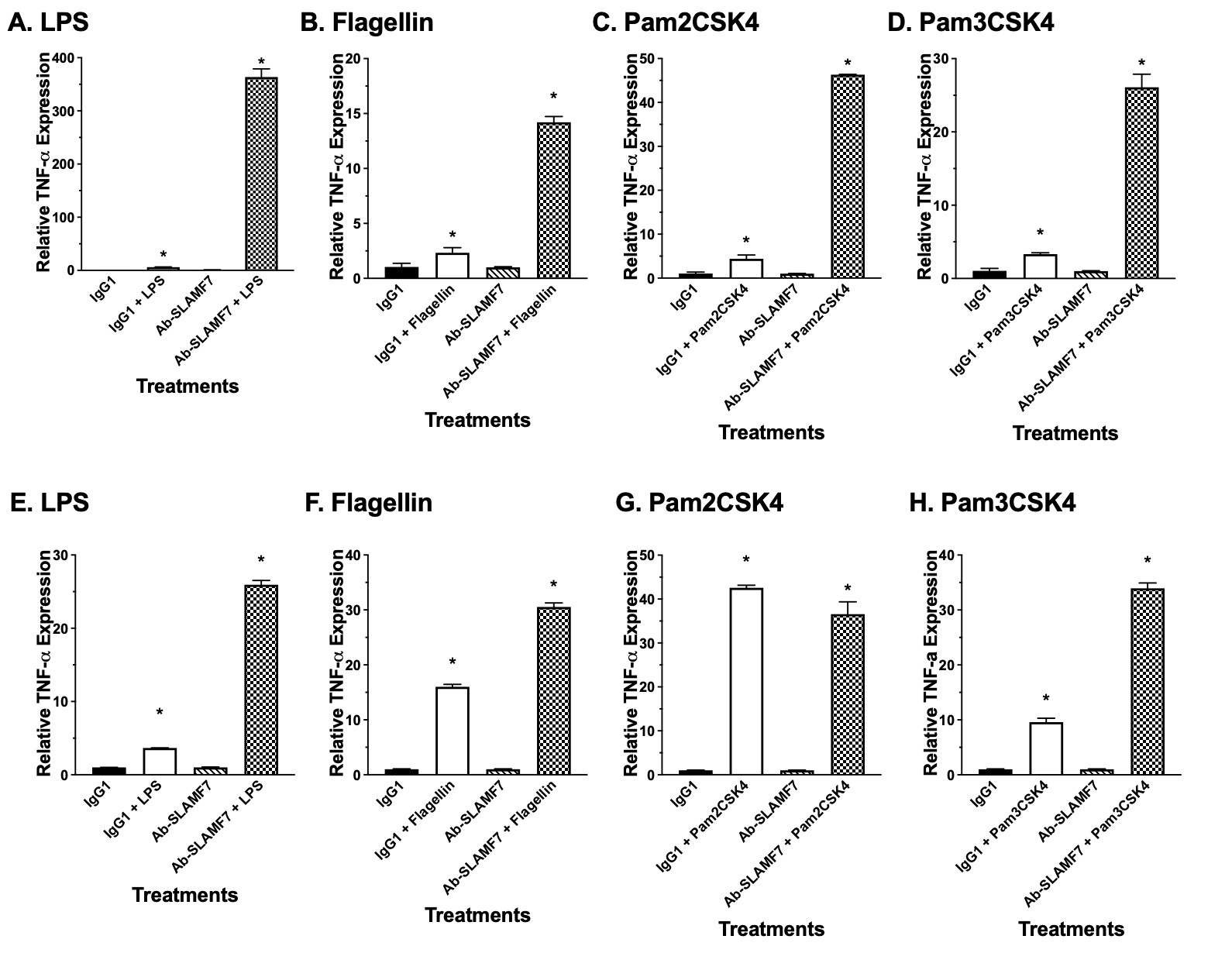


**Fig. S2** Effects of SLAMF7 antibody on TLR-induced TNF-α mRNA levels. Cells were treated with or without TLR ligands in the presence or absence of SLAMF7 antibody. Total RNA isolated after treatments and TNF-α mRNA levels were determined using real-time PCR. Results are expressed as mean ± SD (n = 3). Bars with asterisk indicate significant difference to the un-treated control at *p* ≤ 0.05.  **Panel (A)** LPS **(B)** Flagellin **(C)** Pam2CSK4 **(D)** Pam3CSK4 illustrate effects in u-THP-1. Panel **(E)** LPS **(F)** Flagellin **(G)** Pam2CSK4 **(H)** Pam3CSK4 illustrate effects in d-THP-1.

Figure S3


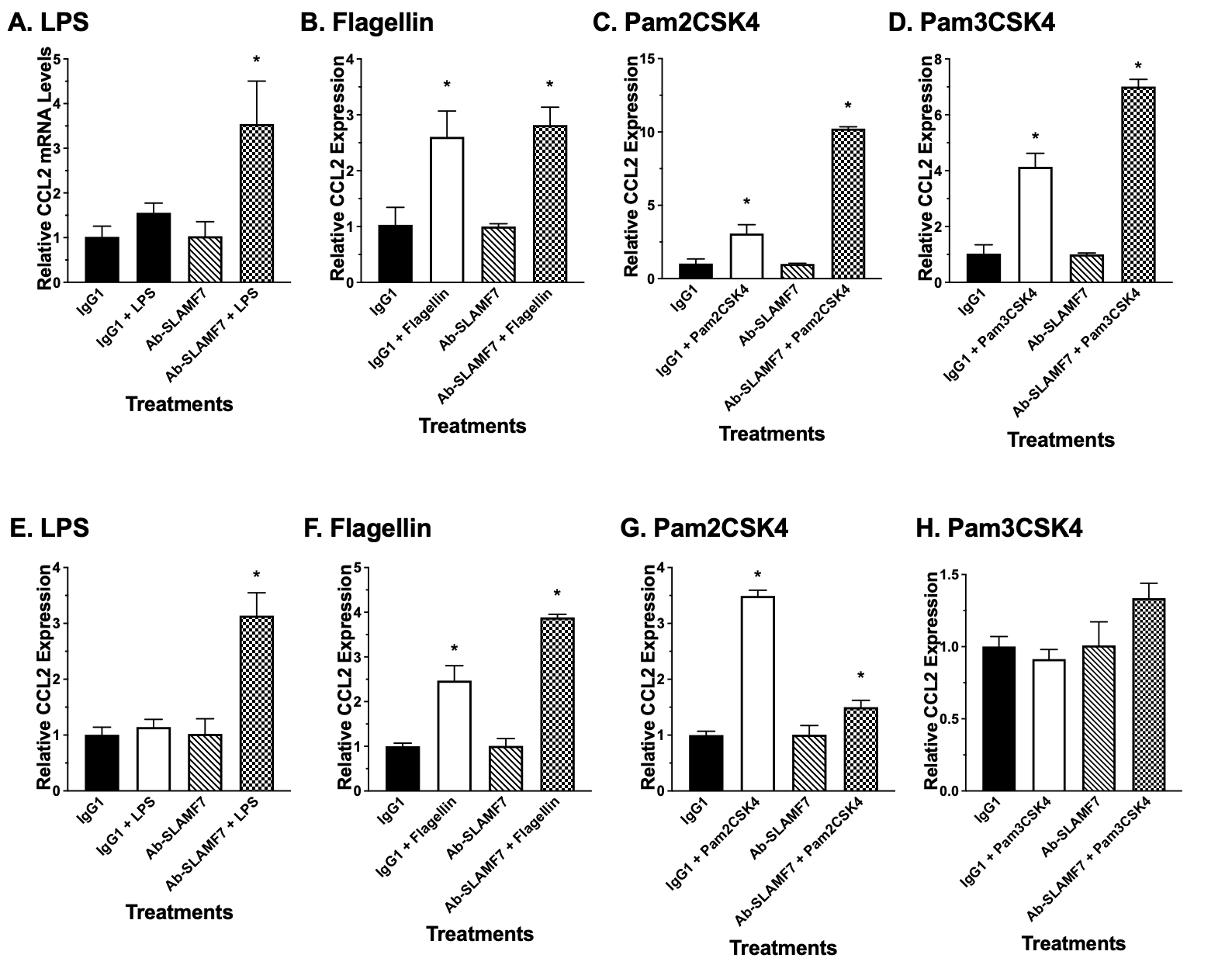


**Fig. S3** Effects of SLAMF7 antibody on TLR-induced CCL2 mRNA levels. Cells were treated with or without TLR ligands in the presence or absence of SLAMF7 antibody. Total RNA isolated after treatments and CCL2 mRNA levels were determined using real-time PCR. Results are expressed as mean ± SD (n = 3). Bars with asterisk indicate significant difference to the un-treated control at *p* ≤ 0.05.  **Panel (A)** LPS **(B)** Flagellin **(C)** Pam2CSK4 **(D)** Pam3CSK4 illustrate effects in u-THP-1. Panel **(E)** LPS **(F)** Flagellin **(G)** Pam2CSK4 **(H)** Pam3CSK4 illustrate effects in d-THP-1.
