# Supplementary material for: Identification and elucidation of cross talk between SLAM Family Member 7 (SLAMF7) and Toll-like receptor (TLR) pathways in monocytes and macrophages
Source: Sci Rep. 2023 Jul 7;13:11007. doi: 10.1038/s41598-023-37040-0 (PMC10329007; doi:10.1038/s41598-023-37040-0)

Figure WB1 Full gel Western Blot for SLAMF5. Lanes 3,4,5,6,7,8 from right was used for Figure 2 A tope panel.

Exposure 1.


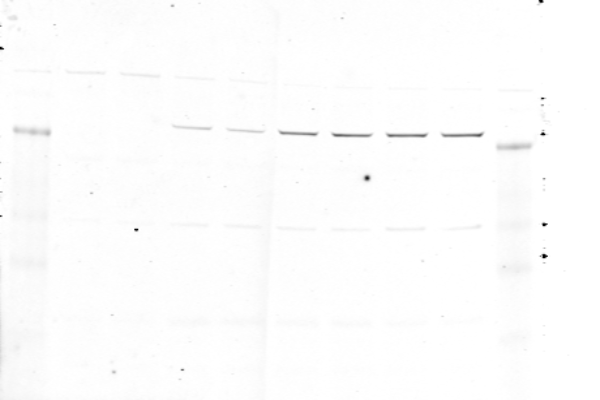


Exposure 2.


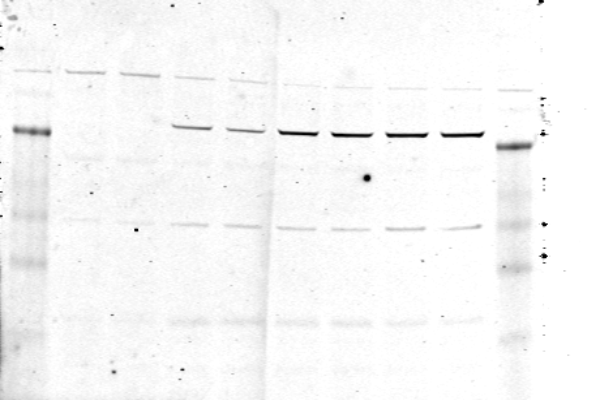


Figure WB2. Full gel Western Blot for Actin. Lanes 2, 3, 4, 5, 6, 7 from left were used for Figure 2 A bottom panel.

Exposure 1.


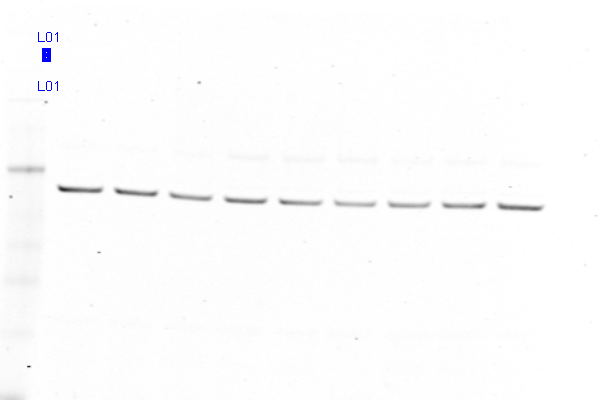


Exposure 2.


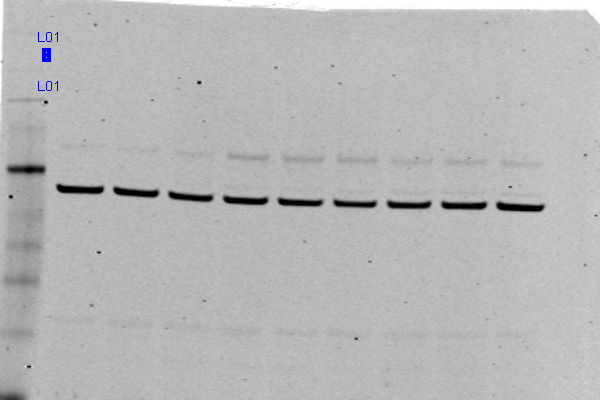


Figure WB3. Full gel image for SLAMF7. Lanes 2, 3, 4 from left was used to generate Figure 2B top panel.

Exposure 1.


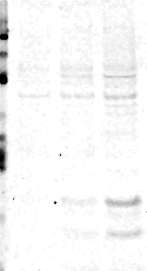


Exposure 2.


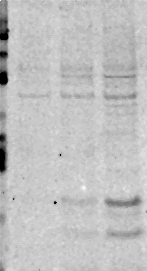


Figure WB4. Full Gel image for actin. Lanes 2,3,4 from left (marker lane) were used to generate Figure 2B bottom panel.

Exposure 1.


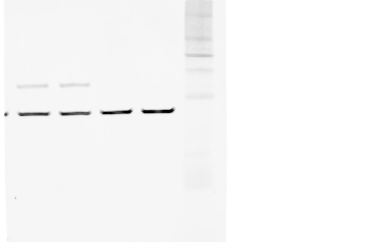


Exposure 2.


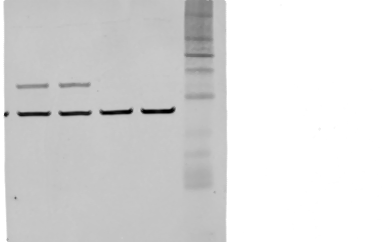


Figure WB5. Full gel Western blot for SLAMF7. Lanes 2-10 from left were used to generate Figure 5 top panel.

Exposure 1.


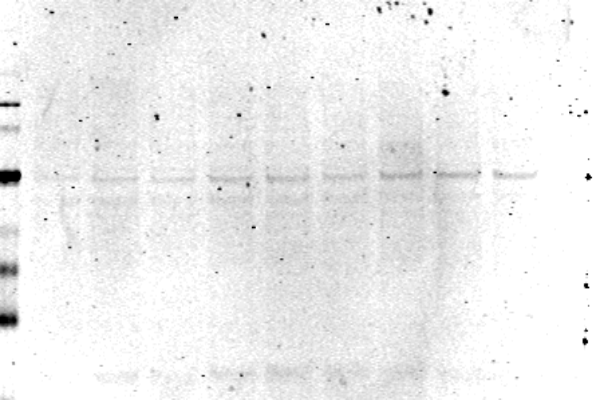


Exposure 2.


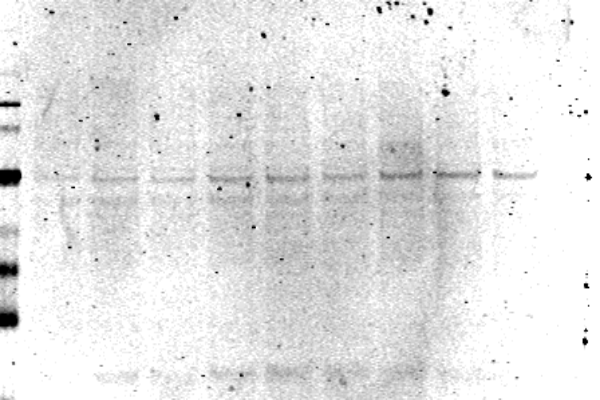


Figure WB6. Full gel image for actin. Lanes 2-10 from left were used to generate Figure 5 bottom panel.

Exposure 1.


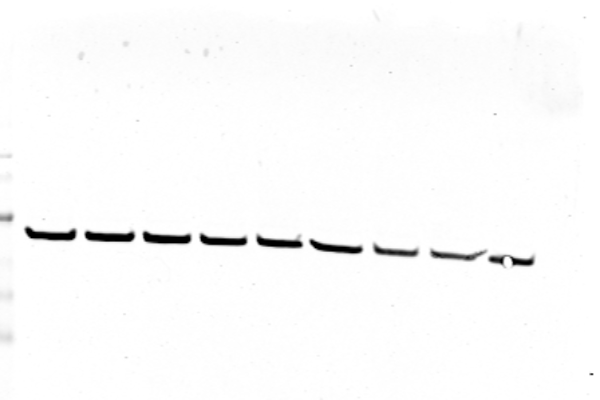


Exposure 2.


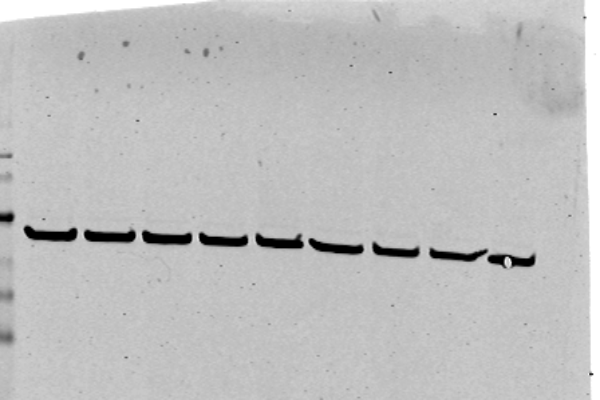

Supplement: Supplementary file 2 — Supplementary Information 1. [file 41598_2023_37040_MOESM2_ESM.docx]
